# Ceftazidime/avibactam resistance is associated with PER-3-producing ST309 lineage in Chilean clinical isolates of non-carbapenemase producing *Pseudomonas aeruginosa*

**DOI:** 10.3389/fcimb.2024.1410834

**Published:** 2024-06-05

**Authors:** Katherine D. Soto, Manuel Alcalde-Rico, Juan A. Ugalde, Jorge Olivares-Pacheco, Valeria Quiroz, Bárbara Brito, Lina M. Rivas, José M. Munita, Patricia C. García, Aniela Wozniak

**Affiliations:** ^1^ Laboratory of Microbiology, Department of Clinical Laboratories; Escuela de Medicina, Pontificia Universidad Católica de Chile, Santiago, Chile; ^2^ Instituto de Ciencias e Innovación en Medicina (ICIM), Facultad de Medicina, Universidad del Desarrollo. Millennium Initiative for Collaborative Research on Bacterial Resistance (MICROB-R), Santiago, Chile; ^3^ Grupo de Resistencia Antimicrobiana en Bacterias Patógenas y Ambientales (GRABPA), Instituto de Biología, Pontificia Universidad Católica de Valparaíso, Valparaíso, Chile; ^4^ Instituto de Biomedicina de Sevilla, Hospital Universitario Virgen Macarena, Consejo Superior de Investigaciones Científicas (CSIC), Universidad de Sevilla, Sevilla, Spain; ^5^ Centro de Investigación Biomédica en Red de Enfermedades Infecciosas, Instituto de Salud Carlos III, Madrid, Spain; ^6^ Center for Bioinformatics and Integrative Biology, Facultad de Ciencias de la Vida, Universidad Andrés Bello, Santiago, Chile; ^7^ Genomics and Resistant Microbes Group (GeRM), Instituto de Ciencias e Innovación en Medicina (ICIM), Facultad de Medicina Clínica Alemana, Universidad del Desarrollo, Santiago, Chile; ^8^ Australian Institute for Microbiology and Infection, Faculty of Science, University of Technology Sydney, Sydney, NSW, Australia; ^9^ Clinical Laboratories Network, Red de Salud UC-CHRISTUS, Santiago, Chile

**Keywords:** *Pseudomonas aeruginosa*, blaPER-3 gene, PER-3 extended-spectrum β-lactamase, ceftazidime/avibactam resistance, mutations conferring CZA resistance

## Abstract

**Introduction:**

Ceftazidime/avibactam (CZA) is indicated against multidrug-resistant *Pseudomonas aeruginosa*, particularly those that are carbapenem resistant. CZA resistance in *P. aeruginosa* producing PER, a class A extended-spectrum β-lactamase, has been well documented *in vitro*. However, data regarding clinical isolates are scarce. Our aim was to analyze the contribution of PER to CZA resistance in non-carbapenemase-producing *P. aeruginosa* clinical isolates that were ceftazidime and/or carbapenem non-susceptible.

**Methods:**

Antimicrobial susceptibility was determined through agar dilution and broth microdilution, while *bla*_PER_ gene was screened through PCR. All PER-positive isolates and five PER-negative isolates were analyzed through Whole Genome Sequencing. The mutational resistome associated to CZA resistance was determined through sequence analysis of genes coding for PBPs 1b, 3 and 4, MexAB-OprM regulators MexZ, MexR, NalC and NalD, AmpC regulators AmpD and AmpR, and OprD porin. Loss of *bla*_PER-3_ gene was induced in a PER-positive isolate by successive passages at 43°C without antibiotics.

**Results:**

Twenty-six of 287 isolates studied (9.1%) were CZA-resistant. Thirteen of 26 CZA-resistant isolates (50%) carried *bla*_PER_. One isolate carried *bla*_PER_ but was CZA-susceptible. PER-producing isolates had significantly higher MICs for CZA, amikacin, gentamicin, ceftazidime, meropenem and ciprofloxacin than non-PER-producing isolates. All PER-producing isolates were ST309 and their *bla*_PER-3_ gene was associated to ISCR1, an insertion sequence known to mobilize adjacent DNA. PER-negative isolates were classified as ST41, ST235 (two isolates), ST395 and ST253. PER-negative isolates carried genes for narrow-spectrum β-lactamases and the mutational resistome showed that all isolates had one major alteration in at least one of the genes analyzed. Loss of *bla*_PER-3_ gene restored susceptibility to CZA, ceftolozane/tazobactam and other β-lactamsin the *in vitro* evolved isolate.

**Discussion:**

PER-3-producing ST309 *P. aeruginosa* is a successful multidrug-resistant clone with *bla_PER-3_* gene implicated in resistance to CZA and other β-lactams.

## Introduction

Carbapenem-resistant *Pseudomonas aeruginosa* is a global public health problem. Ceftazidime/avibactam (CZA) is a combination of a third-generation cephalosporin and an inhibitor of classes A, C, and some class D β-lactamases (Papp-Wallace et al., 2020). CZA was approved by the Food and Drug Administration (FDA) in 2015 and has become an alternative for treating infections caused by carbapenem-resistant bacteria (Papp-Wallace et al., 2020). Multiple mechanisms of resistance to CZA have been described in *P. aeruginosa.* It has been reported that resistance to CZA may be acquired through mutation of penicillin-binding proteins (PBPs) (Castanheira et al., 2019), overexpression and/or structural modifications in the Ω-loop region of the chromosomally-encoded AmpC β-lactamase (Ruedas-López et al., 2022). Experimental *in vitro* evolution assays and *in vivo* evidence showed that mutations related to overexpression and/or structural modification of MexAB-OprM and MexCD-OprJ efflux pump are associated with resistance to CZA (Chalhoub et al., 2018; Sanz-García et al., 2018; Castanheira et al., 2019). These mutations often involve inactivation of MexR, NalC, or NalD which are regulators of MexAB-OprM, and cause the overexpression of these efflux pumps resulting in 2- to 16- fold MIC increases to all the pump substrates compared to baseline levels (Adewoye et al., 2002). OprD porin inactivation was observed in CZA resistant clinical isolates of *P. aeruginosa* (Zamudio et al., 2019). Some variants of extended-spectrum β-lactamases (ESBLs) have also been associated with CZA resistance, including VEB-1, VEB-9, GES-26, GES-19, GES-7, SHV-2, SHV-5 and PER-1 (Kazmierczak et al., 2018; Khan et al., 2019; Mendes et al., 2019). PER (*Pseudomonas*-Extended-Resistant) is a class A ESBL that confers resistance to penicillins, broad-spectrum cephalosporins, and aztreonam (Philippon et al., 2016). In total, 16 variants of PER have been described. PER-1 is the most prevalent PER type in Europe and Asia, whereas PER-2 is frequent in South America (Xie et al., 2016). PER-3 has been reported in *Aeromonas* sp (Xie et al., 2016), and *P. aeruginosa* (Mojica et al., 2023), among other species. The *bla*_PER_ gene was reported to be in the chromosome (*Proteus mirabilis*) and in plasmids (*P. aeruginosa, P. mirabilis, Aeromonas sp and Enterobacter cloacae)* (Xie et al., 2016). Although avibactam efficiently inhibits most class A β-lactamases, its efficacy against PER-type enzymes is reduced compared to other β-lactamases of the same class. The hydrophobicity of PER’s active site hampers the binding of water molecules that are crucial for the acylation step required for an efficient enzymatic inhibition (Ruggiero et al., 2019). PER-mediated resistance to CZA in *P. aeruginosa* has been demonstrated *in vitro* by introducing *bla*_PER-1_ in the reference strain PAO1 (Poirel et al., 2022; Ortiz de la Rosa et al., 2019). However, evidence of the contribution of PER to CZA resistance in clinical isolates of *P. aeruginosa* is scarce.

In 2018 we obtained the first CZA resistant clinical isolate of *P. aeruginosa* that produced PER-3. During the COVID-19 pandemic, the use of carbapenems and CZA increased significantly and carbapenem-resistant *P. aeruginosa* increased as well (Allel et al., 2023). In this pandemic period, several PER-producing *P. aeruginosa* clinical isolates were obtained. This is of major concern because the effectiveness of CZA, approved in 2019 for clinical use in Chile, is being compromised. Our objective was to analyze the contribution of PER to CZA resistance through determining the prevalence of CZA resistance and *bla*_PER_ gene in non-carbapenemase producing clinical isolates of *P. aeruginosa*, which were non-susceptible to carbapenems and/or ceftazidime, and to characterize the genetic context of *bla*_PER_ gene. Moreover, we analyzed the contribution of PER to CZA resistance in a clinical isolate that was induced to lose the *bla_PER_* gene and restoration of β-lactam susceptibility was analyzed thereof.

## Materials and methods

### Clinical isolates

The first clinical isolate of PER-producing *P. aeruginosa* (MF-1) in the University Hospital of Pontificia Universidad Católica de Chile was obtained in 2018 from blood cultures of a 14-year-old male patient. MF-1 was resistant to CZA (MIC >16 μg/ml), according to Clinical Laboratory Standards Institute (CLSI 2023) breakpoints (Clinical and Laboratory Standards Institute, 2023). CZA was not administered to the patient, but he had received meropenem and vancomycin because of polymicrobial infection with *Enterococcus* and ESBL-producing *Enterobacteriaceae*.

In the present work, we screened for all non-carbapenemase producing clinical isolates of *P. aeruginosa* with a non-susceptible phenotype to ceftazidime and/or carbapenems (imipenem and meropenem) obtained in the Laboratory of Microbiology of the University Hospital between January-December 2021. The total number of *P. aeruginosa* isolates obtained during this period was 1314, and 287 isolates fulfilled the above-mentioned criteria. The source of the isolates were respiratory specimens (119), urine (91), wounds (33), blood (19), tissue (11), skin (6), abscesses (5), peritoneal fluid (2), and cerebrospinal fluid (1). All 287 isolates had a negative carba-NP test performed according to CLSI recommendations (Clinical and Laboratory Standards Institute, 2023). All isolates were screened for the carbapenemase genes *bla*_KPC_, *bla*_OXA-48_, *bla*_NDM_, *bla*_IMP_, *bla*_SPM_, *bla*_GES_, *bla*_VIM_ through conventional PCR as described (Wozniak et al., 2021). Only the first isolate of each patient that fulfilled the above-described criteria was included. Surveillance isolates were excluded from this study. Bacterial species were identified through Matrix Assisted Laser Desorption - Time of Flight Mass Spectrometry (MALDI-TOF) (Bruker-Daltonics, Bremen, Germany). All the 287 isolates were screened for *bla*_PER_ and *bla*_VEB_ genes through PCR using primers and conditions as described (Fazeli et al., 2015). This study was approved by the Ethics and Biosafety Committee of Pontificia Universidad Católica de Chile.

### *In vitro* evolution of *bla*_PER-3_-positive isolates

To induce the loss of *bla*_PER-3_, isolate MF-1 (the first PER-producing isolate obtained in 2018) was grown in 25 mL of tryptic soy broth (TSB) (Beckton Dickinson) without antibiotics, and incubated at 37°C or 43°C with constant shaking. The culture broth was daily sub-cultured (dilution 1/100) in fresh medium for 14 days, and each day, 10 µl from the culture broth were seeded in PIA plates (*Pseudomonas* Isolation Agar) and incubated for 24 h at 37°C. Every following day, a total of 16 colonies were screened through PCR for detection of the *bla*_PER_ gene. No PER-negative colonies were obtained at 37°C. One PER-negative colony was obtained at 43°C. This *in vitro* evolved isolate was named MF-2.

### Antimicrobial susceptibility testing

#### Agar dilution method

Susceptibility to CZA, ceftazidime, imipenem, meropenem, cefoperazone/sulbactam, piperacillin/tazobactam, ciprofloxacin, and amikacin of all isolates was determined through the agar dilution method as per CLSI 2023 recommendations and breakpoints (Clinical and Laboratory Standards Institute, 2023). Briefly, bacterial suspensions were adjusted to McFarland 0.5 and dispensed in the seeding-tray wells of a Cathra Replicator System (32 wells). To determine minimum inhibitory concentration (MIC), Mueller-Hinton agar plates (BBL™ BD, Sparks, MD) supplemented with doubling antibiotic concentrations were seeded using 1 mm-pins. To determine the MIC of CZA, the doubling dilution range for ceftazidime spanned from 0.125 μg/ml to 16 μg/ml, with a fixed avibactam concentration of 4 μg/ml. Plates were incubated at 37°C for 18–20 h before reading. Breakpoints used for cefoperazone/sulbactam were those indicated in the package insert of Sulperazon (Pfizer). All assays were performed in triplicate. All antimicrobial compounds were obtained from USP (United States Pharmacopeia), except for ceftazidime that was obtained from Sigma-Aldrich, and avibactam that was provided by Pfizer in the context of a Pure Compound Grant (PCG) ID#75444555 awarded to AW.

#### Broth microdilution method

Susceptibility of MF-1 and its respective *in vitro* evolved counterpart MF-2, was determined through broth microdilution using SensiTitre Antimicrobial Susceptibility Testing System DKMGN according to manufacturer`s instructions (Thermo Fisher Scientific, United States). Susceptibility of all PER-positive isolates to aztreonam and ceftolozane/tazobactam was analyzed through broth microdilution. MIC values were determined after incubation for 18–20 h at 37°C. Susceptibility categorization was performed using the breakpoints suggested by CLSI 2023 (Clinical and Laboratory Standards Institute, 2023). Quality control (QC) tests were performed with reference standard strains *P. aeruginosa* ATCC 27853 and *Escherichia coli* ATCC 25922. QC MIC values for all standard strains were within acceptable ranges.

### Whole-genome sequencing and bioinformatic analysis

Isolate MF-1 was sequenced using long and short read approaches (Illumina and Oxford Nanopore, respectively). Long reads were obtained in a GridION Flow cell at the Garvan Institute of Medical Research (Australia). Short read library preparation was performed using Hackflex protocol (Gaio et al., 2022), and sequencing was conducted through Illumina MiSeq technology at the University of Technology Sydney (Australia). MF-1 genome was assembled using Raven, Flye, and Canu (Murigneux et al., 2020). Finally, assemblies were consolidated using Trycycler, in which short reads were used to polish long reads (Wick et al., 2021). MF-2 and all 14 PER-positive isolates were sequenced through a short read approach. Briefly, sample library was prepared using the Illumina DNA Prep kit and IDT 10 bp UDI indices and sequenced on an Illumina NextSeq-2000. The assembled genomes were annotated using Bakta (Schwengers et al., 2021), and antimicrobial resistance genes were predicted using AMRFinder (Feldgarden et al., 2019). To identify single nucleotide polymorphisms (SNPs) as well as structural differences between parental MF-1 and *in vitro* evolved MF-2 genomes we used the circularized genome as a reference and mapped the short reads using Breseq (Deatherage and Barrick, 2014). Multi-Locus Sequence Typing (MLST) was performed using the Galaxy-Australia platform (Galaxy Australia (Australian Biocommons and partners), [[NoYear]]). Circular genome visualization was performed using pyCirclize (Shimoyama, 2022). The non-synonymous polymorphisms of genes associated with mutational resistome of *P. aeruginosa* (López-Causapé et al., 2017) were identifed by calling SNPs with Snippy v4.6.0 software (https://github.com/tseemann/snippy), mapping the trimmed raw reads of each bacterial isolate with respect the PAO1 reference genome (NC_002516.2) (Delgado-Valverde et al., 2024). In addition, amino acid sequences were compared with that of susceptible isolates of the same STs using ClustalW. Amino acid substitutions that were present in PAO1 and/or in the susceptible isolate of the same ST were considered polymorphisms and were not reported as SNPs. The genomes of susceptible isolates were obtained from PATRIC database and their Genome IDs were 1402550.3 (ST309), 652611.13 (ST253, PA14 genome), 1447536.3 (ST395), 1402496.3 (ST235) and 1163395.3 (ST41). The impact of amino acid substitutions in protein functionality was analyzed with SIFT algorithm (Sorting Intolerant From Tolerant) allowing a prediction of functional impact of mutations as “deleterious” (score ≤ 0.05) or “neutral” (score >0.05) (Vaser et al., 2016).

## Results

### Susceptibility to CZA and *bla*_PER_ gene occurrence

A total of 287 non-carbapenemase-producing *P. aeruginosa* isolates that were non-susceptible to ceftazidime and/or carbapenems, were included in the study. All isolates had a negative PCR for carbapenemase genes. Twenty-six of them (9.1%) were resistant to CZA (MIC≥16 μg/ml), as determined through agar dilution. Thirteen of the 26 CZA-resistant isolates (50%) carried the *bla*_PER_ gene. Isolate N° 1224 carried *bla*_PER_ gene but was susceptible to CZA (MIC=4 μg/ml). The prevalence of *bla*_PER_ among the cohort analyzed was 4.8% (14/287), whereas 92.8% (13/14) of PER-positive isolates were CZA-resistant. To compare MIC values between PER-positive and PER-negative isolates, the following isolate after each PER-positive isolate was selected. PER-positive isolates had MIC values for CZA, amikacin, gentamicin, ceftazidime, meropenem and ciprofloxacin that were significantly higher than PER-negative isolates (**Table 1**). In contrast, MICs for cefoperazone/sulbactam, piperacillin/tazobactam, colistin and imipenem were not significantly different between PER-positive and PER-negative isolates (**Table 1**). All PER-positive isolates were further analyzed for their susceptibility to ceftolozane/tazobactam and aztreonam through broth microdilution. Thirteen out of fourteen PER-positive isolates were resistant to ceftolozane/tazobactam, whereas isolate 1224 was intermediately resistant (MIC = 8 μg/ml), and all isolates were resistant to aztreonam.

**Table 1:** MICs of antimicrobials for PER-negative and PER-positive isolates.

MIC (μg/ml) and category of PER-negative isolates										
ID N°	AMK	GEN	CAZ	CZA	C/S	P/T	IPM	MPN	CIP	COL
740	4 (S)	≤4 (S)	4 (S)	8 (S)	32 (I)	32 (I)	≥16 (R)	≥16 (R)	0.5 (S)	<2 (I)
898	8 (S)	≤4 (S)	16 (I)	8 (S)	32 (I)	128 (R)	2 (S)	≤1 (S)	0.5 (S)	<2 (I)
1074	4 (S)	≤4 (S)	1 (S)	1 (S)	≤16 (S)	≤16 (S)	4 (I)	≤1 (S)	≤0.25 (S)	2 (I)
1164	4 (S)	≤4 (S)	32 (R)	4 (S)	64 (R)	128 (R)	≤1 (S)	4 (I)	≤0.25 (S)	<2 (I)
1172	64 (R)	≥16 (R)	8 (S)	16 (R)	≤16 (S)	≤16 (S)	≥16 (R)	≥16 (R)	0.5 (S)	<2 (I)
1189	8 (S)	≤4 (S)	8 (S)	8 (S)	32 (I)	32 (I)	≥16 (R)	≥16 (R)	2 (R)	2 (I)
1228	4 (S)	≤4 (S)	2 (S)	2 (S)	≤16 (S)	≤16 (S)	4 (I)	≤1 (S)	≤0.25 (S)	2 (I)
1264	4 (S)	≤4 (S)	1 (S)	1 (S)	≤16 (S)	≤16 (S)	4 (I)	≤1 (S)	≤0.25 (S)	2 (I)
1275	4 (S)	≤4 (S)	4 (S)	1 (S)	≤16 (S)	≤16 (S)	4 (I)	≤1 (S)	≥4 (R)	2 (I)
1285	≤2 (S)	≤4 (S)	1 (S)	1 (S)	≤16 (S)	≤16 (S)	4 (I)	≤1 (S)	1 (I)	2 (I)
1360	8 (S)	≤4 (S)	4 (S)	4 (S)	≤16 (S)	≤16 (S)	4 (I)	≤1 (S)	≤0.25 (S)	2 (I)
1373	8 (S)	8 (I)	8 (S)	8 (S)	32 (I)	32 (I)	≥16 (R)	≥16 (R)	2 (R)	2 (I)
1487	8 (S)	≤4 (S)	4 (S)	4 (S)	≤16 (S)	≤16 (S)	8 (R)	4 (I)	≥4 (R)	2 (I)
1674	≤2 (S)	≤4 (S)	0.5 (S)	0.5 (S)	≤16 (S)	≤16 (S)	≥16 (R)	2 (S)	≤0.25 (S)	2 (I)
MIC (μg/ml) and category of PER-positive isolates												
ID N°	AMK	GEN	CAZ	CZA	C/S	P/T	IPM	MPN	CIP	COL	C/T	AZT
738	8 (S)	≤4 (S)	≥128 (R)	>16 (R)	32 (I)	128 (R)	≥16 (R)	≥16 (R)	≥4 (R)	<2 (I)	≥32 (R)	≥32 (R)
896	4 (S)	≤4 (S)	≥128 (R)	16 (R)	≤16 (S)	32 (I)	≥16 (R)	≥16 (R)	2 (R)	2 (I)	≥32 (R)	≥32 (R)
1073	32 (I)	≥16 (R)	≥128 (R)	16 (R)	32 (I)	32 (I)	≥16 (R)	≥16 (R)	≥4 (R)	2 (I)	≥32 (R)	≥32 (R)
1161	32 (I)	≥16 (R)	≥128 (R)	16 (R)	≤16 (S)	≤16 (S)	≥16 (R)	8 (R)	2 (R)	2 (I)	≥32 (R)	≥32 (R)
1169	32 (I)	≥16 (R)	≥128 (R)	16 (R)	≤16 (S)	≤16 (S)	8 (R)	2 (S)	1 (I)	≥4 (R)	16 (R)	≥32 (R)
1186	64 (R)	≥16 (R)	≥128 (R)	16 (R)	≤16 (S)	≤16 (S)	≥16 (R)	8 (R)	2 (R)	2 (I)	≥32 (R)	≥32 (R)
1224	32 (I)	≥16 (R)	≥128 (R)	4 (S)	≤16 (S)	≤16 (S)	≥16 (R)	8 (R)	≤0.25 (S)	<2 (I)	8 (I)	≥32 (R)
1263	64 (R)	≥16 (R)	≥128 (R)	16 (R)	≤16 (S)	≤16 (S)	≥16 (R)	≥16 (R)	≥4 (R)	2 (I)	≥32 (R)	≥32 (R)
1270	64 (R)	≥16 (R)	≥128 (R)	16 (R)	≤16 (S)	≤16 (S)	≥16 (R)	≥16 (R)	≥4 (R)	2 (I)	≥32 (R)	≥32 (R)
1282	4 (S)	≤4 (S)	≥128 (R)	>16 (R)	64 (R)	128 (R)	≥16 (R)	≥16 (R)	1 (I)	<2 (I)	≥32 (R)	≥32 (R)
1359	32 (I)	≥16 (R)	≥128 (R)	>16 (R)	≤16 (S)	≤16 (S)	≥16 (R)	4 (I)	2 (R)	2 (I)	≥32 (R)	≥32 (R)
1368	32 (I)	≥16 (R)	≥128 (R)	>16 (R)	≤16 (S)	64 (R)	≥16 (R)	≥16 (R)	≥4 (R)	≥4 (R)	≥32 (R)	≥32 (R)
1486	32 (I)	≥16 (R)	≥128 (R)	>16 (R)	≤16 (S)	32 (I)	≥16 (R)	8 (R)	1 (I)	<2 (I)	≥32 (R)	≥32 (R)
1673	32 (I)	≥16 (R)	≥128 (R)	>16 (R)	≤16 (S)	32 (I)	≥16 (R)	≥16 (R)	≥4 (R)	2 (I)	≥32 (R)	≥32 (R)
P value	0.0003	0.0018	0.0019	<0.0001	0.6776	0.7036	0.4815	0.0159	0.0044	0.4815		
Signif.	***	**	**	****	NS	NS	NS	*	**	NS		

MICs were determined through agar dilution and interpreted according to CLSI 2023 guidelines. PER-negative isolates are a subsample of the 273 PER-negative isolates selected as the following isolate to each PER-positive isolate. AMK, amikacin; GEN, gentamicin; CAZ, ceftazidime; CZA, ceftazidime/avibactam; C/S, cefoperazone/sulbactam; P/T, piperacillin/tazobactam; IPM, imipenem; MPN, meropenem; CIP, ciprofloxacin; COL, colistin; C/T, ceftolozane/tazobactam; AZT, aztreonam. Intermediate (I) and resistant (R) MICs values were shaded grey. The difference between percentages of non-susceptible (categories R and I) and susceptible (S) isolates in PER-negative versus PER-positive isolates for each antimicrobial was analyzed using Fisher´s exact test with a confidence interval of 95%; P value and significance (Signif.) is shown; NS, not significant. Statistical analyses were done using GraphPad Prism version 9.0 (La Jolla, California, USA). *, P<0.05; **, P<0.005; ***, P<0.0005; ****, P<0.00005.

### Genomic analysis of PER-positive and PER-negative isolates

**Figure 1:**
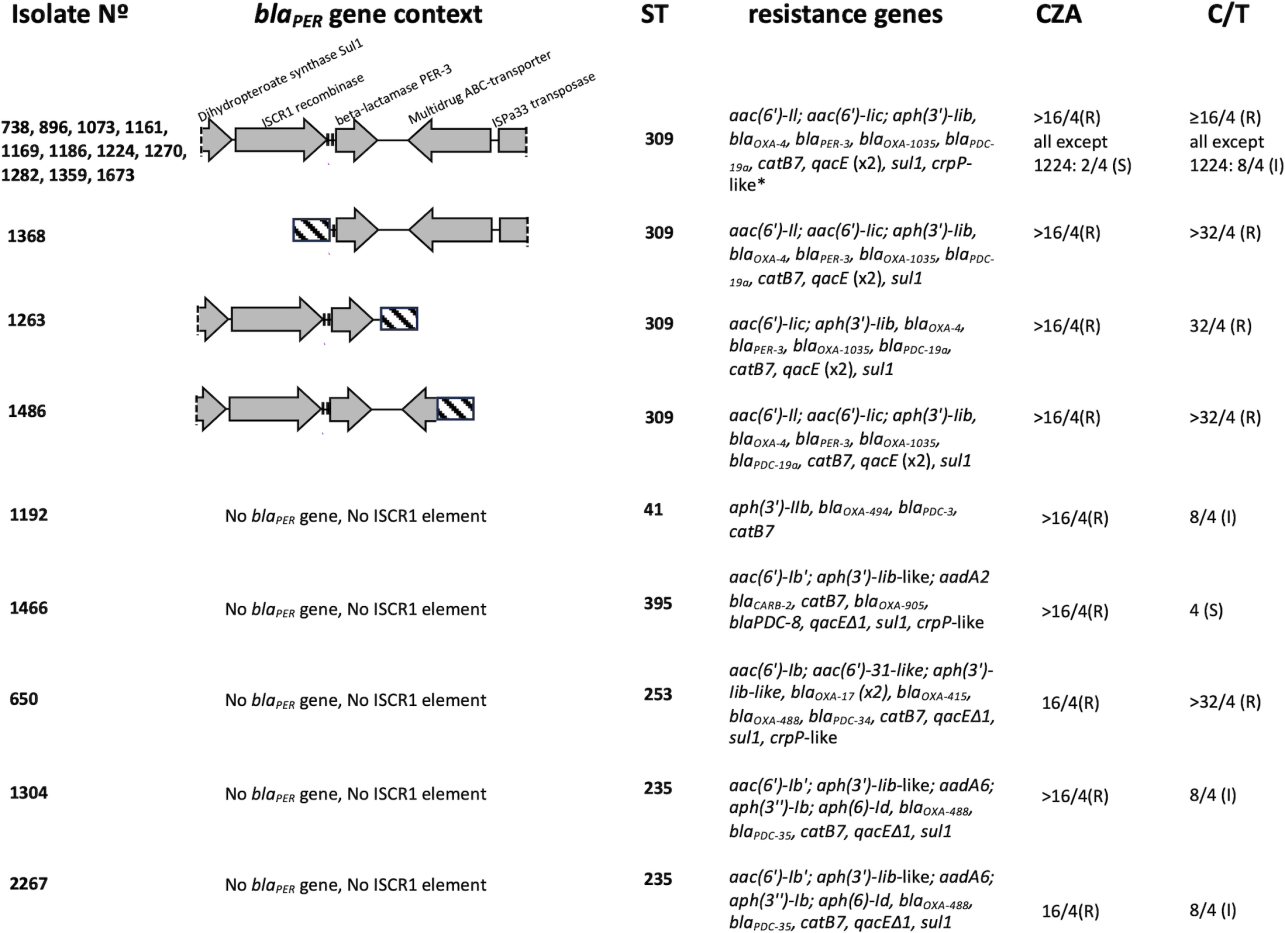
Genetic characteristics of PER-positive and PER-negative isolates. All PER-positive isolates except 1368 had an ISCR1 element upstream *blaPER-3* gene composed by the ISCR1 gene coding for a site-specific recombinase and two adjacent 28bp-target sequences (vertical bars). *detected only in isolate 1673. Striped bars represent sequences that are non-homologous among isolates. CZA, ceftazidime-avibactam; C/T, ceftolozane-tazobactam.

All the 14 PER-positive isolates were sequenced through short reads WGS. MLST analysis classified them as sequence type (ST) 309. The *bla*_PER_ gene of all the isolates was PER-3 type. PlasmidSpades was run for all PER-positive isolates and no plasmids were found. In all isolates except 1368, the *bla*_PER-3_ gene was associated with an insertion sequence formed by an *ISCR1* gene that encodes a site-specific recombinase and two adjacent 28 bp-target sequences (direct repeats) that are recognized by the ISCR1 recombinase (Arduino et al., 2002; Partridge and Hall, 2003). Isolate 1368 had its ISCR1 recombinase gene in a different contig. Downstream the *bla*_PER-3_ gene all isolates except 1263 and 1486 carried genes coding for an ABC transporter ATP-binding protein and an ISPa33-family transposase (**Figure 1**). According to AMRFinder, all 14 PER-positive isolates carried the resistance genes *bla*_PER-3_ and *bla*_OXA-4_ (**Figure 1**). All isolates carried the same alleles for the intrinsic *P. aeruginosa* β-lactamases AmpC and OXA-50: the *bla_PDC_* (AmpC) allele was PDC-19a and the *bla_OXA-50_* allele was OXA-1035. Mutations in chromosomal genes known to be associated to CZA resistance were analyzed and are referred as mutational resistome (López-Causapé et al., 2017) (**Table 2**). Genes analyzed were those coding for PBPs 1b, 3 and 4, MexAB-OprM regulators MexZ, MexR, NalC and NalD, AmpC regulators AmpD and AmpR, and OprD porin. Sequences were compared to wild type sequences of PAO1 and to CZA susceptible isolates of the same STs. Several amino acid substitutions were found in PER-positive ST309 isolates respect to PAO1, but most of them were also found in the CZA susceptible ST309 strain, suggesting that they are ST-related polymorphisms, and they were not reported here. Major alterations not observed in the CZA susceptible strain i.e., insertions, premature stop codons, amino acid substitutions, or frameshift mutations were detected in MexZ of isolates 1673, 1169, 1263, 1270 and 1368. MexR, NalD and NalC had no major alterations except for isolate 1263 that had the amino acid substitution T11N in NalD (**Table 2**). Penicillin-binding proteins PBP1b, PBP3 and PBP4, and regulators of AmpC expression AmpD and AmpR, and OprD porin had no major alterations in none of the PER-positive isolates. The CZA susceptible isolate 1224 had no major alterations in the genes analyzed (**Table 2**). Moreover, this isolate had an intact *bla*_PER-3_ gene and promoter and has the same resistance genes as the other PER-positive isolates. The analysis of mutational resistome showed that this isolate had amino acid changes in PmrA and CprS (E191K and D141N respectively), a response regulator and a sensor kinase respectively, of two-component systems involved in lipopolysaccharide modification causing polymyxin resistance (Fernández et al., 2012). Regarding resistance to non-β-lactam antibiotics, notably, all PER-positive isolates had two genes for aminoglycoside modifying enzymes that may account for their resistance to amikacin and gentamicin (**Figure 1**). Regarding genes associated with fluoroquinolone resistance, one isolate had an amino acid change in GyrA (D87N) and 4 isolates had an amino acid change in ParC (data not shown). Besides this, 3 isolates had a *crpP*-like gene that is known to contribute to ciprofloxacin resistance (Chávez-Jacobo et al., 2018) (**Figure 1**).

**Table 2:** Mutation analysis of genes associated to CZA resistance.

Mutational resistome of PER-positive isolates												
ID N°	MexZ	MexR	NalC	NalD	PBP1b	PBP3	PBP4	AmpD	AmpR	OprD	GyrA	ParC
738	–	–	–	–	–	–	–	–	–	–	D87N	S87L
896	–	–	–	–	–	–	–	–	–	–	–	–
1073	–	–	–	–	–	–	–	–	–	–	–	–
1161	–	–	–	–	–	–	–	–	–	–	–	–
1169	dup K6	–	–	–	–	–	–	–	–	–	–	–
1186	–	–	–	–	–	–	–	–	–	–	–	–
1224	–	–	–	–	–	–	–	–	–	–	–	–
1263	dup K6	–	–	T11N*	–	–	–	–	–	–	–	S87L
1270	fs D209	–	–	–	–	–	–	–	–	–	–	S87L
1282	–	–	–	–	–	–	–	–	–	–	–	–
1359	–	–	–	–	–	–	–	–	–	–	–	–
1368	fs D209	–	–	–	–	–	–	–	–	–	–	S87L
1486	–	–	–	–	–	–	–	–	–	–	–	–
1673	//84	–	–	–	–	–	–	–	–	–	–	–
Mutational resistome of CZA resistant PER-negative isolates												
1192	–	R63H	–	–	–	–	T73P*	–	–	–	–	–
1466	–	–	–	fs V151	–	–	G117S	–	–	fs Q402	–	S87L
650	T5A*	–	–	–	E70D	–	–	–	–	fs Q402	–	S87L
1304	211 W	–	–	//35	–	T295S	–	E120K*	–	//57	–	S87L
2267	211 W	–	–	//35	–	T295S	–	E120K*	–	//57	–	S87L

Sequences of genes associated with CZA resistance i.e. mexR, mexZ, nalD, ponB, ftsI, dacB, ampD, oprD coding for MexR, MexZ, NalD, PBP1b, PBP3, PBP4, AmpD and OprD respectively, were compared to sequences of a susceptible reference isolate of the same ST obtained from PATRIC database. Sequences of genes associated to quinolone resistance were also analyzed (gyrA and parC). Genome IDs used were: ST309: 1402550.3; ST253 (PA14 genome) 652611.13; ST395: 1447536.3; ST235: 1402496.3; ST41: 1163395.3. dup: duplication; //: stop codon at indicated position; fs: frameshift at indicated position; -, no mutation found respect to susceptible isolate; *SIFT scores predicted as deleterious for protein functionality (≤0.05).

Five PER-negative CZA resistant isolates were analyzed through short reads WGS. Their sequence types were ST41, ST235 (two isolates), ST395 and ST253. The *bla_PDC_* alleles were PDC-3, PDC-35 (two isolates), PDC-8 and PDC-34, respectively. PER-negative isolates carried genes for narrow-spectrum β-lactamases: isolate 650 had two copies of *bla_OXA-17_* (OXA-10-like) and one copy of *bla_OXA-415_* (OXA-2-like); isolate 1466 had a *bla_CARB-2_* gene; isolates 1304, 2267 and 650 carried *bla_OXA-488_* (OXA-50-like) (**Figure 1**). In contrast to PER-positive isolates, the mutational resistome of PER-negative isolates showed that all isolates had one major alteration in at least one of the genes analyzed (**Table 2**). Isolates 1304 and 2267 had a mutation in the stop codon of MexZ, such that the protein was 36 amino acids longer than the wild type one and isolate 650 had the amino acid substitution T5A. MexR had the amino acid substitution R63H in isolate 1192. NalD had a frameshift mutation in position V151 in isolate 1466, and it was truncated in isolates 1304 and 1267. In contrast to PER-positive isolates, PER-negative isolates had major alterations in penicillin-binding proteins as specified in **Table 2**. Isolates 1304 and 2267 had the amino acid change E120K in AmpD (**Table 2**). Only isolate 1192 had a wild type OprD, the other four isolates had a frameshift mutation or were truncated.

### *In vitro* evolved isolate that lost the *bla*_PER-3_ gene became susceptible to CZA and other β-lactams

To investigate the contribution of PER-3 to β-lactam resistance, loss of the *bla*_PER-3_ gene was induced in the PER-3-positive isolate MF-1, by successive subcultures at 37°C or 43°C without antibiotics. Colony PCR was performed to determine the presence/absence of the *bla*_PER-3_ gene. No PER-negative colonies were obtained at 37°C after 14 days of subcultures. In contrast, a PER-negative colony was obtained the first day of subculture at 43°C. Loss of *bla*_PER-3_ gene was determined by a negative amplification in the *bla_PER_*-targeted PCR. This *in vitro* evolved isolate was designated MF-2. Susceptibility of the parental isolate and it’s *in vitro* evolved isogenic counterpart was determined through broth microdilution and is shown in **Table 2**. Loss of *bla_PER_* restored the susceptibility to ceftazidime, CZA and ceftolozane/tazobactam in isolate MF-2. Furthermore, MICs of aztreonam and cefepime decreased from resistant to intermediately resistant (**Table 3**). In contrast, MICs of piperacillin/tazobactam and carbapenems as well as the other antimicrobials tested were not affected upon loss of *bla*_PER-3_ (**Table 3**).

**Table 3:** Antimicrobial susceptibility profile of parental PER-positive isolate MF-1 and its *in vitro* evolved PER-negative counterpart MF-2.

	MIC (μg/ml) (category)	
Antimicrobial	MF-1 (PER+)	MF-2 (PER-)
amikacin	32 (I)	32 (I)
gentamicin	>16 (R)	>16 (R)
piperacillin/tazobactam	32 (I)	32 (I)
**cefepime**	**32 (R)**	**16 (I)**
**ceftazidime**	**>128 (R)**	**8 (S)**
**ceftazidime/avibactam**	**>16 (R)**	**4 (S)**
**ceftolozane/tazobactam**	**>32 (R)**	**1 (S)**
**aztreonam**	**>32 (R)**	**16 (I)**
meropenem	16 (R)	16 (R)
imipenem	16 (R)	16 (R)
colistin	2 (I)	2 (I)
ciprofloxacin	>4 (R)	>4 (R)

Categories were assigned according to CLSI 2023 guidelines: R, resistant; I, intermediately resistant; S, susceptible. MIC, Minimum Inhibitory Concentration. All antimicrobials were evaluated through broth microdilution. Antimicrobials that changed category in the *in vitro* evolved isolate are shown in bold letters.

### Comparative genomic analysis of parental and *in vitro* evolved isolate

**Figure 2:**
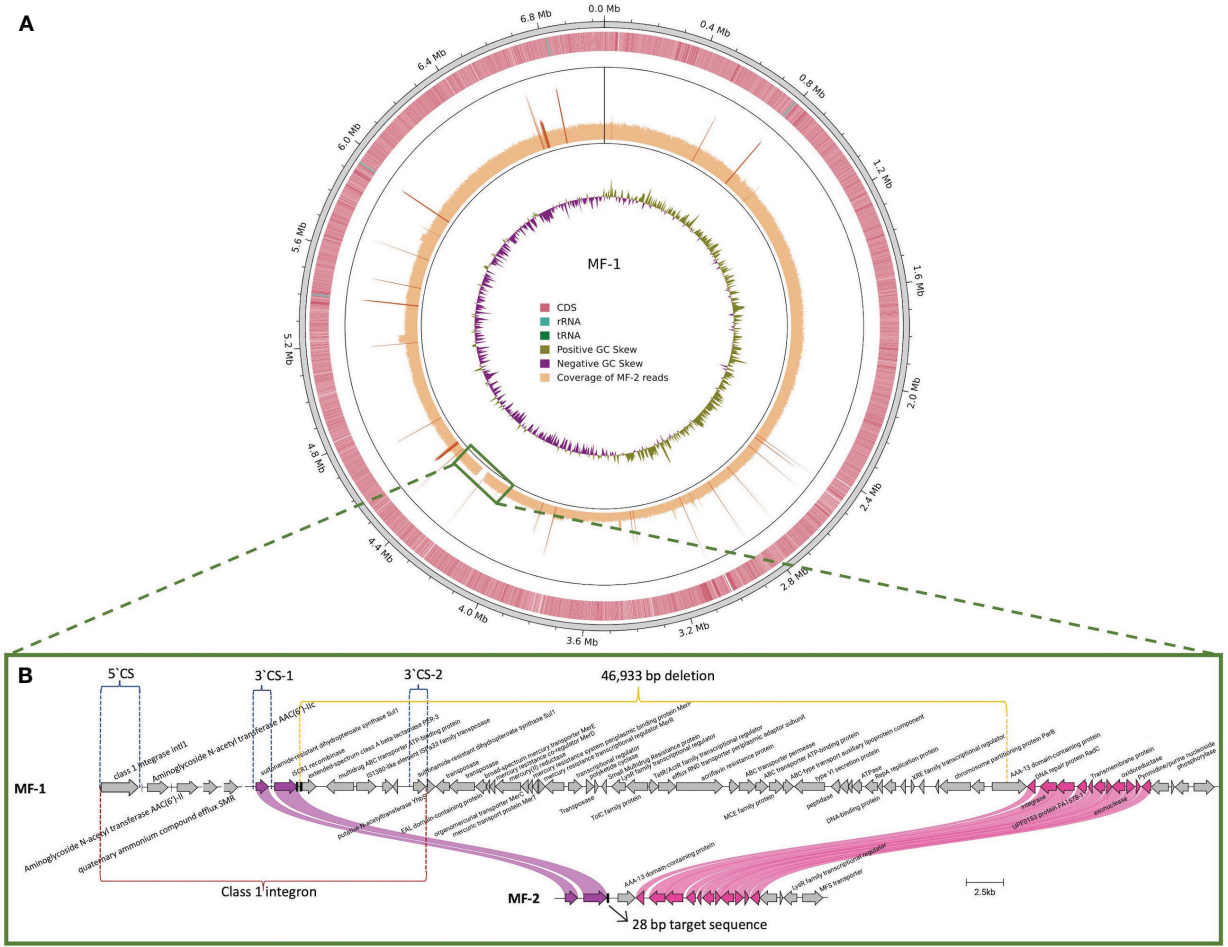
Genomic regions involved in loss of *bla_PER-3_* gene in isolate MF-1 and *in vitro* evolved isolate MF-2. **(A)** MF-1 chromosome was obtained through hybrid alignment of long+short reads. Coverage of MF-2 short reads mapped against MF-1 chromosome is shown. The green square indicates the uncovered region. **(B)** The genetic content of the 46,933 bp deletion is shown. The *bla*_PER-3_ gene is embedded in a class 1 integron (red lines). The 3`CS (conserved segment) of the integron is duplicated as 3`CS-1 and 3`CS-2 and the 5`CS includes the class 1 integrase (blue lines). Purple and pink curved lines show the upstream and downstream limits of the deletion. Black vertical bars: 28 bp-target sequences of ISCR1.

MF-1 was sequenced through short and long reads WGS to fully close its genome. The hybrid assembly of MF-1 produced a single circularized contig of 7,043,257 bp that corresponded to the bacterial chromosome (**Figure 2A**). The *bla*_PER-3_ gene was associated to an ISCR1 element, the same found in the other PER-positive isolates (**Figures 1**, **2B**). Sequence analysis showed that *bla*_PER-3_ gene was embedded in a particular type of class 1 integron harboring two copies of the 3`CS (Conserved Sequence, containing the *sul1* gene), with *bla*_PER-3_ gene located between both 3`CS copies (3`CS-1 and 3`CS-2) (**Figure 2B**) (Partridge and Hall, 2003). MF-2 was sequenced through short reads WGS. To compare MF-1 and MF-2 genomes, MF-2 reads were mapped against the closed chromosome of MF-1. MF-2 short reads covered the MF-1 genome entirely, except for a 46,933 bp fragment (**Figure 2A**), that was lost in MF-2 isolate. This DNA fragment included the *bla*_PER-3_ gene and several genes encoding efflux pumps, ABC transporters and transcriptional regulators, among other traits (**Figure 2B**). The complete list of genes included in the 47 kb fragment is shown in **Supplementary Figure S1**. MF-2 harbored the same resistance genes as MF-1, except for the absence of *bla*_PER-3_. In addition, mapping analysis identified 44 SNPs between MF-1 and MF-2: 6 intergenic, 1 nonsense, 12 non-synonymous, 23 in pseudogenes, and 2 synonymous (**Supplementary Figure S2**). However, none of these polymorphisms was identified in genes previously associated with CZA resistance or their regulatory elements. Sequences of MF-1 and MF-2 were deposited in the Genbank database with accession numbers CP117527 and NZ_JARFMG000000000 respectively.

## Discussion

The prevalence of *bla*_PER_ among the isolates studied here was 4.8% (14/287), whereas it was 15.2% (14/92) among ceftazidime non-susceptible isolates. In contrast, the prevalence of *bla*_PER_ among ceftazidime non-susceptible isolates reported in other regions is considerably higher, namely 29% in Europe (Mendes et al., 2019), 86% in Turkey (Aktaş et al., 2005), and 81% in Iran (Haghighi and Reza Goli, 2022). Notably, we did not detect VEB and GES, which are highly prevalent in regions like Asia-Pacific and Thailand, with frequencies of 80% and 100%, respectively (Karlowsky et al., 2018).

In this work 13 of 14 PER-positive isolates were CZA resistant. Moreover, the *in vitro* evolved isolate that lost *bla*_PER-3_ became susceptible to CZA, ceftolozane/tazobactam and other antipseudomonal β-lactams. Although the SNP analysis showed that *in vitro* evolved isolate MF-2 did not acquire mutations in genes associated to CZA resistance, it is reasonable to think that other mutations induced by high temperature could be contributing to susceptibility to CZA. The evidence presented in this work about clinical isolates reinforces the results obtained with isogenic mutants expressing PER that indicate that PER-3 is the main responsible for the resistance to CZA and may compromise the success of new lines of therapies.

CZA resistance in PER-negative isolates is most likely associated with narrow-spectrum β-lactamases and/or mutations in genes associated to CZA resistance *i.e. mexR, mexZ, nalD, ponB, ftsI, dacB, ampD, oprD*. OXA-488 and CARB-2 have been reported in CZA resistant *P. aeruginosa* clinical isolates (Sid Ahmed et al., 2022; Babouee Flury et al., 2023). OXA-17 is an OXA-10-like oxacillinase that has been associated with resistance to CZA and ceftolozane/tazobactam (Sid Ahmed et al., 2022). The mutational resistome of PER-negative isolates showed that they had mutations in AmpC, efflux pump regulators, PBPs and OprD porin; some of them have been reported in CZA resistant *P. aeruginosa* isolates that did not have other carbapenemases (Babouee Flury et al., 2023). Isolate 1192 has an AmpC variant not associated with CZA resistance and has no narrow-spectrum β-lactamases but it has a mutation in PBP4 that was predicted as deleterious. PBP4 mutants were shown to have increased expression of AmpC which may account for its CZA resistance (Zamorano et al., 2010). These findings are in accordance with the multifactorial nature of CZA resistance, particularly in *P. aeruginosa*; several determinants acting together is needed to achieve resistance, e.g., narrow-spectrum β-lactamases or carbapenemases, increased efflux pump expression, and altered permeability of antimicrobials (Wang et al., 2020; Babouee Flury et al., 2023). The fact that the CZA susceptible isolate 1224 had the same resistance genes and similar mutational resistome as the other PER-positive CZA resistant isolates, indicates that other mechanisms may be related to CZA susceptibility. For instance, mutations in PmrA and CprS that modulate the composition of lipopolysaccharide in the outer membrane, could confer hyper-susceptibility to CZA in isolate 1224.

The role of ISCR1 in *bla_PER_* mobilization has been reported in a clinical isolate of *Vibrio cholerae* (Wu et al., 2015). The excision of the 47 kb fragment in MF-2 occurred between the two 28 bp target sequences of ISCR1, suggesting that this recombinase is likely involved in this deletion. The loss of *bla*_PER-3_ in MF-1 by the action of ISCR1 recombinase suggests that the stress conditions imposed by high temperature (43°C) may have induced the IS-dependent mobilization of resistance genes (Guerin et al., 2009). Although further research needs to be carried out, it is remarkable that stability of chromosomal resistance genes, which are usually considered more stable than plasmid-located ones, could be compromised in response to temperature-induced stress.

The fact that all PER-3-producing isolates belong to the same clone (ST309) and have the same resistance genes and genetic environment of the *bla*_PER-3_ gene, suggests that ST309 is a relevant vehicle of PER-3. Moreover, our results show that PER-3-producing ST309 lineage is associated with resistance to amikacin, meropenem, ciprofloxacin, and ceftolozane/tazobactam. Intensive use of CZA may select for the PER-3 variant, increasing CZA resistance levels further, and exacerbating the lack of options for the treatment of infections caused by multidrug resistant bacteria. This is particularly serious in our setting because there are no therapeutic options for infections caused by organisms like this; cefiderocol or meropenem/vaborbactam are not available in Chile yet. ST309 is not among the top high-risk clones described worldwide that include ST111, ST235, ST175, ST375 (del Barrio-Tofiño et al., 2020). However, ST309 is now being considered a newly emerging high-risk clone: it has been reported in severe infections associated with resistance to CZA and ceftolozane/tazobactam in Mexico, United States (Khan et al., 2019), and Brazil (Fonseca et al., 2022). It is of major importance to know the main circulating STs, resistance mechanisms and genetic platforms to detect the potential emergence of new high-risk clones and predict what could happen in other regions.

## Data availability statement

The datasets presented in this study can be found in online repositories. The names of the repository/repositories and accession number(s) can be found below:https://www.ncbi.nlm.nih.gov/genbank/, CP117527 https://www.ncbi.nlm.nih.gov/genbank/, NZ_JARFMG000000000.

## Ethics statement

The studies involving humans were approved by Comité Ético Científico de Ciencias de la salud UC. The studies were conducted in accordance with the local legislation and institutional requirements. The human samples used in this study were acquired from primarily isolated as part of your previous study for which ethical approval was obtained. Written informed consent for participation was not required from the participants or the participants’ legal guardians/next of kin in accordance with the national legislation and institutional requirements.

## Author contributions

KS: Data curation, Formal analysis, Methodology, Project administration, Validation, Writing – original draft. MA-R: Conceptualization, Formal analysis, Investigation, Methodology, Project administration, Resources, Software, Supervision, Validation, Writing – review & editing. JU: Data curation, Formal analysis, Investigation, Methodology, Resources, Software, Visualization, Writing – review & editing. JO-P: Formal analysis, Investigation, Methodology, Resources, Supervision, Writing – review & editing. VQ: Data curation, Investigation, Methodology, Writing – review & editing. BB: Data curation, Formal analysis, Funding acquisition, Investigation, Methodology, Software, Visualization, Writing – review & editing. LR: Data curation, Investigation, Methodology, Project administration, Resources, Supervision, Writing – review & editing. JM: Conceptualization, Formal analysis, Funding acquisition, Investigation, Project administration, Resources, Supervision, Writing – review & editing. PG: Conceptualization, Formal analysis, Funding acquisition, Project administration, Resources, Supervision, Validation, Writing – review & editing. AW: Conceptualization, Data curation, Formal analysis, Funding acquisition, Investigation, Methodology, Project administration, Resources, Supervision, Validation, Writing – original draft.
