# Supplementary material for: Ceftazidime/avibactam resistance is associated with PER-3-producing ST309 lineage in Chilean clinical isolates of non-carbapenemase producing Pseudomonas aeruginosa
Source: Front Cell Infect Microbiol. 2024 Jun 5;14:1410834. doi: 10.3389/fcimb.2024.1410834 (PMC11188487; doi:10.3389/fcimb.2024.1410834)
Supplement: Supplementary file 1 [file DataSheet_1.docx]

**Genes in the 47kb fragment that was lost in MF-2**

The genes in the 47kb deletion include: multidrug ABC transporter operons, ISPa33 transposase and other transposases, dihydropteroate synthase (*sul1*), N-acetyl-transferase, EAL-domain containing signaling protein, mercury-resistance operon, polyketide cyclase, small-multidrug-resistance transporter protein, transcriptional regulators, TolC family protein, TetR/AcrR transcriptional regulator, acriflavine resistance multidrug efflux system (*acrA/acrB*), chromosome partitioning protein (*parB*), peptidases, ATPases, TypeVI secretion protein, replication proteins, MCE family protein, and several hypothetical proteins.
